# *Cry1**Δ**11* mutation induces ADHD-like symptoms through hyperactive dopamine D1 receptor signaling

**DOI:** 10.1172/jci.insight.170434

**Published:** 2023-08-22

**Authors:** Dengfeng Liu, Zhengyu Xie, Panyang Gu, Xiangyu Li, Yichun Zhang, Xinying Wang, Zhiheng Chen, Suixin Deng, Yousheng Shu, Jia-Da Li

**Affiliations:** 1 Furong Laboratory, Center for Medical Genetics, School of Life Sciences, Central South University, Changsha, Hunan, P. R. China.; 2 School of Life Sciences, Changsha Medical University, Changsha, Hunan, P. R. China.; 3 Department of Pediatrics, the Third Xiangya Hospital, Central South University, Changsha, P. R. China.; 4 Department of Neurology, Huashan Hospital, State Key Laboratory of Medical Neurobiology, Institute for Translational Brain Research, MOE Frontiers Center for Brain Science, Fudan University, Shanghai, China.; 5 Hunan Key Laboratory of Animal Models for Human Diseases, Changsha, Hunan, P. R. China.; 6 Hunan Key Laboratory of Medical Genetics, Changsha, Hunan, P. R. China.

**Keywords:** Neuroscience, Psychiatric diseases

## Abstract

Attention-deficit hyperactivity disorder (ADHD) is a highly heritable neurodevelopmental disorder that affects approximately 5.3% of children and approximately 2.5% of adults. There is an intimate relationship between ADHD and sleep disturbance. Specifically, individuals carry a mutation in the core circadian gene *CRY1* (c. 1657 + 3A > C), which results in the deletion of exon 11 expression in the CRY1 protein (CRY1Δ11), causing them to exhibit typical ADHD symptoms. However, the underlying mechanism is still elusive. In this study, we demonstrate that *Cry1Δ11* (c. 1717 + 3A > C) mice showed ADHD-like symptoms, including hyperactivity, impulsivity, and deficits in learning and memory. A hyperactive cAMP signaling pathway was found in the nucleus accumbens (NAc) of *Cry1Δ11* mice. We further demonstrated that upregulated c-Fos was mainly localized in dopamine D1 receptor-expressing medium spiny neurons (DRD1-MSNs) in the NAc. Neuronal excitability of DRD1-MSNs in the NAc of *Cry1Δ11* mice was significantly higher than that of WT controls. Mechanistically, the CRY1Δ11 protein, in contrast to the WT CRY1 protein, failed to interact with the Gαs protein and inhibit DRD1 signaling. Finally, the DRD1 antagonist SCH23390 normalized most ADHD-like symptoms in *Cry1Δ11* mice. Thus, our results reveal hyperactive DRD1 signaling as an underlying mechanism and therapeutic target for ADHD induced by the highly prevalent *CRY1Δ11* mutation.

## Graphical abstract

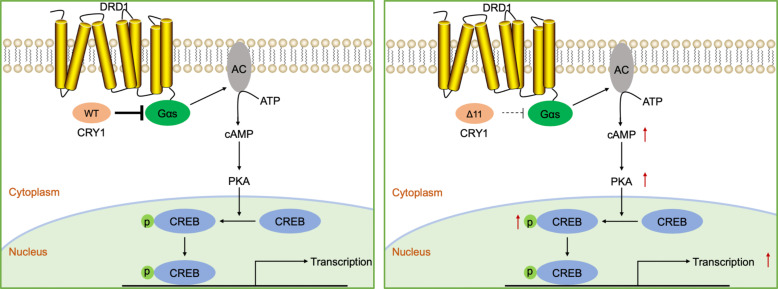

## Introduction

Attention-deficit and hyperactivity disorder (ADHD) is a highly heritable neurodevelopmental disorder characterized by frequent, pervasive, and impairing symptoms of inattention and/or hyperactivity/impulsivity (1). The prevalence of ADHD in children and adolescents is around 5.3%, whereas its prevalence in adults is about 2.5% (1). ADHD can increase the risk of other psychiatric disorders, educational and occupational failure, accidents, criminality, social disability, and addictions, causing a serious burden on personal life, family, and society (2). The etiology of ADHD is complex, involving genetics, mild brain injury, and adverse social and family environments. The heritability of ADHD is estimated to be 0.75–0.91, but the specific genetic mode and pathogenesis are still unclear (2, 3).

There is an intimate connection between ADHD and sleep disturbance. Children with ADHD have significantly more impaired sleep compared with their healthy peers (4). ADHD has been associated with increased sleep duration and increased daytime sleepiness. Furthermore, individuals with ADHD may be more likely to have an evening circadian preference and often take longer to fall asleep (4, 5). Abnormal circadian rhythms of melatonin secretion have also been observed in ADHD patients (5).

Sleep is regulated by a homeostatic process and a circadian rhythm. Circadian rhythms with an approximately 24-hour periodicity are present in various biological processes at the molecular and behavioral levels (6), and the processes are controlled by a cell-autonomous oscillator, i.e., circadian clock (7). The basic machinery of the circadian clock is a transcription- and translation-based negative feedback loop. In mammals, CLOCK and BMAL1 form a heterodimer to transactivate *PERIOD1–3* (*PER1–3*) and *CRYPTOCHROME1–2* (*CRY1–2*) through binding to the E-box element within their promoters (7, 8). The PER and CRY proteins dimerize and repress activity of CLOCK/BMAL1 and, subsequently, inhibit their own transcription (9). Furthermore, REV-ERBs and retinoic acid receptor-related orphan receptor (ROR) proteins regulate *BMAL1* transcription negatively and positively, respectively, forming an additional regulatory loop (9).

Genetic studies have revealed that a 3′-UTR polymorphism of the *CLOCK* gene (rs1801260) is associated with evening preference, and the T-allele of rs1801260 has also been found to increase the risk for adult ADHD in certain ethnic populations (10). Moreover, while *BMAL1* and *PER2* gene expression has circadian rhythmicity in normal individuals, this circadian rhythmicity is lost in ADHD patients (11). Interestingly, a recent animal study demonstrated that *PER1b*-deficient zebrafish and *PER1*-KO mice display hyperactive, impulsive-like, and cognitive deficit-like behaviors (12). Moreover, hyperactivity was also found in *ClockΔ19* mutant mice and *Rev-erba*–KO mice (13, 14).

Recently, a mutation in the core circadian clock gene *CRY1* (c. 1657 + 3A > C) has been identified in a hereditary form of delayed sleep phase disorder (DSPD) (15). This allele, with a frequency of up to 0.44% (0–3.346%, gnomAD), leads to deletion of exon 11 expression, which encodes 24 aa residues in the C-terminal region of the CRY1 protein (CRY1Δ11) (15). Reverse phenotyping revealed *CRY1Δ11* to be significantly associated with ADHD (16). Nevertheless, the pathologic mechanism underlying induction of ADHD by the *CRY1Δ11* mutation is unclear.

In this study, we generated and behaviorally characterized *Cry1Δ11*-knockin mice. We show that *Cry1Δ11*-knockin mice mimic ADHD-like behaviors and demonstrate that hyperactive dopamine D1 receptor (DRD1) signaling may be the underlying pathological mechanism and therapeutic target for ADHD induced by the *CRY1Δ11* mutation.

## Results

### Generation of Cry1Δ11-knockin mice.

**Figure 1:**
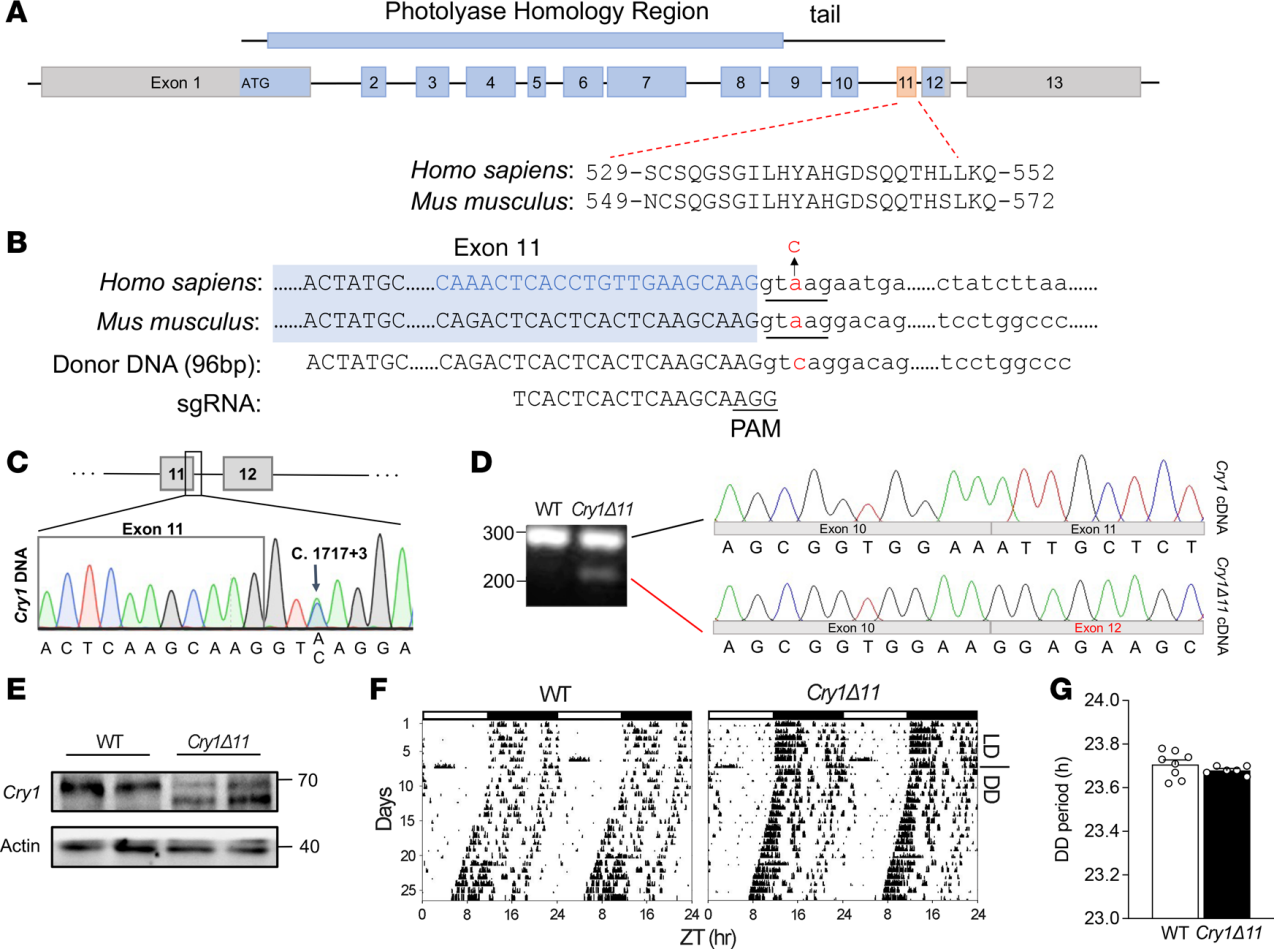
Generation of *Cry1Δ11*-knockin mice. (**A**) Schematic representation of mouse Cry1 protein (top), genomic structure (middle), and aa sequence encoded by exon 11 of human or mouse *Cry1* gene (bottom). (**B**) Comparison of the nucleotide sequences at the junction of exon 11 and intron 11 of human and mouse *Cry1* genes. The mutation (A > C) is labeled in red; the 96-bp sequences of donor DNA and sgRNA used for gene editing are shown at the bottom. (**C**) Sanger sequencing of the junction between exon 11 and intron 11 confirmed the existence of the desired nucleotide modification (c. 1717 + 3A > C). Mouse tail genomic DNA was amplified by PCR and the product was analyzed by Sanger sequencing. (**D**) Sanger sequencing of the cDNA confirmed deletion of exon 11 in the *Cry1* (c. 1717 + 3A > C) mutant mouse. RNA samples extracted from the livers of WT and mutant mice were reverse transcribed to cDNA. The cDNA was amplified by PCR and the products from mutant mice were cloned into a pMD20-T vector by TA cloning (Takara, 6028). Sanger sequencing showed that the top band corresponded to the WT *Cry1*, whereas the bottom band lacked exon 11. (**E**) Western blot confirmed that the c. 1717 + 3A > C mutation resulted in a truncated Cry1 protein. Proteins from the livers of WT and mutant mice were subjected to Western blot with a Cry1 Ab. (**F**) Representative double-plotted actograms of WT and *Cry1Δ11* mice. (**G**) The free-running periods of WT (*n* = 8) and *Cry1Δ11* mice (*n* = 6); *P* > 0.05, unpaired Student’s *t* test. Data are presented as mean ± SEM.

In humans, the *CRY1* (c. 1657 + 3A > C) mutation leads to mis-splicing, skipping exon 11. To determine whether mutation of the corresponding nucleotide sequence in mice (*Cry1* c. 1717 + 3A > C) may be able to mimic the human phenotype, we first compare the aa sequences encoded by exon 11 of the human and mouse *CRY1* genes. As shown in Figure 1A, exon 11 of human and mouse *CRY1* genes encodes a conserved 24-aa sequence. Furthermore, the 5 nucleotides at the 5′-splicing site where the mutation is located are identical in humans and mice (i.e., GTAAG, Figure 1B). Fortunately, there is a protospacer-adjacent motif (PAM, i.e., AGG) around the mutation site (Figure 1B). We therefore injected a sgRNA and donor DNA (Figure 1B) together with Cas9 mRNA into embryonic day 0.5 oocytes taken from C57BL6 mice. After screening the offspring, we identified several strains of mice with the desired nucleotide change (Figure 1C). Reverse transcription PCR (RT-PCR) plus Sanger sequencing confirmed deletion of exon 11 in the knockin mice, denoted as *Cry1Δ11* mice (Figure 1D). Western blotting with a Cry1-specific Ab showed a band of approximately 70 kDa in WT mice, whereas 2 bands were identified in the *Cry1Δ11* mice (Figure 1E), supporting the production of a truncated Cry1Δ11 protein.

As *CRY1Δ11* was initially identified as the causative mutation of a familial-delayed sleep disorder, we monitored the wheel-running activity of *Cry1Δ11* mice and their WT littermate controls. Animals were housed individually in cages equipped with running wheels. After continuous monitoring of wheel running activities for 2–3 weeks under a 12-hour light/dark (LD) schedule, mice were switched to constant darkness (DD) for 3–4 weeks. Unexpectedly, there was no significant difference in the free-running periods under DD between WT and *Cry1Δ11* mice (Figure 1, F and G).

### Cry1Δ11 mice exhibited hyperactivity and deficits in learning and memory.

As a core circadian gene, expression of *Cry1* varied with a daily cycle. As shown in Supplemental Figure 1; supplemental material available online with this article; https://doi.org/10.1172/jci.insight.170434DS1, expression of *Cry1* in the prefrontal cortex (PFC), nucleus accumbens (NAc), and the hippocampus at zeitgeber time 14 (ZT14) was higher than at ZT02 (ZT is measured in hours after the light has been turned on in a LD cycle). We then performed a battery of ADHD-relevant behavioral tests with *Cry1Δ11* mice and their littermate WT controls during 2 different time periods (ZT02–04 and ZT14–16).

**Figure 2:**
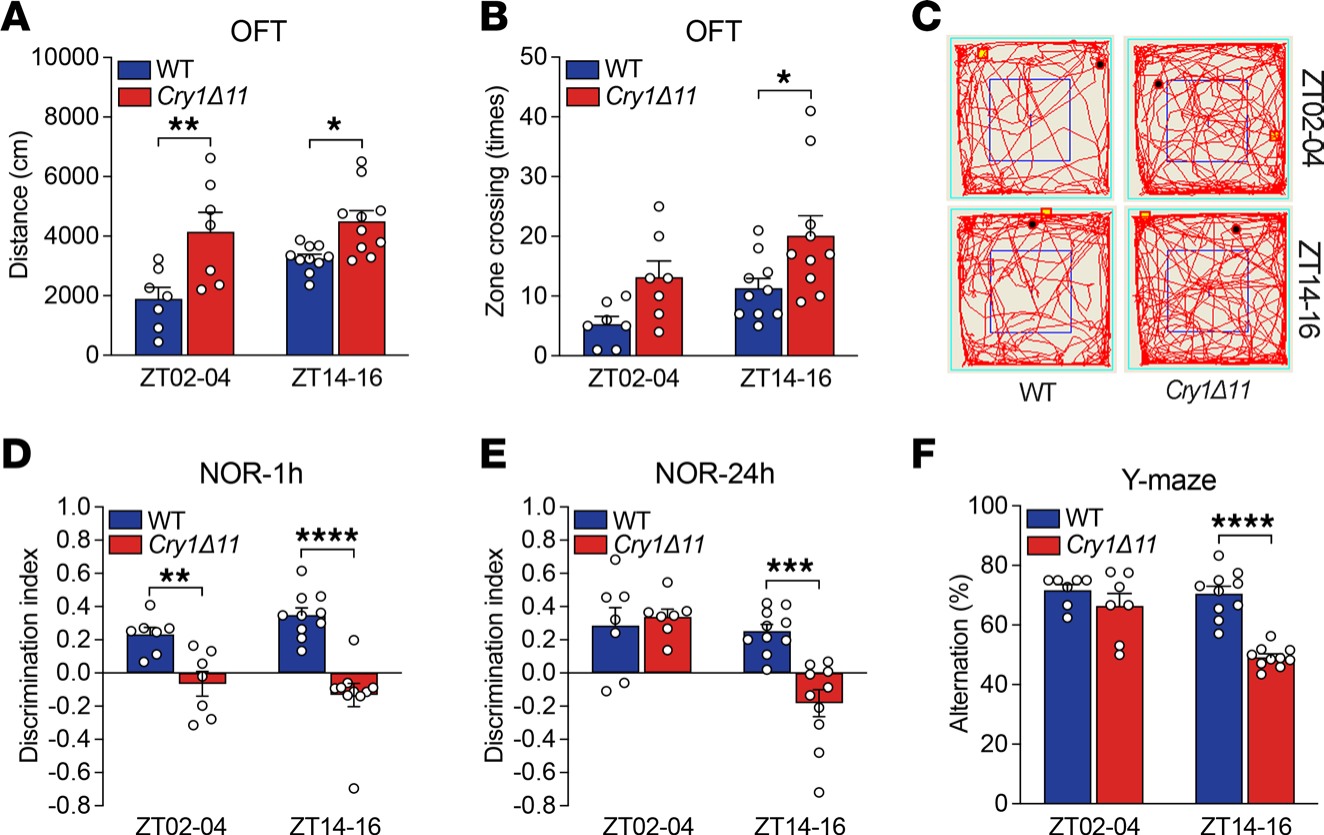
*Cry1Δ11* mice exhibited hyperactivity and deficits in learning and memory. (**A**) *Cry1Δ11* mice travelled significantly longer distances than WT controls in the OFT. Data are presented as mean ± SEM. *n* = 7–10 mice/genotype/time point; Genotype: F (1, 30) = 20.18; *P < 0.05, **P < 0.01, 2-way ANOVA followed by Bonferroni’s *t* test. (**B**) The number of zone crossings in the OFT test was significantly higher in *Cry1Δ11* mice. Data are presented as mean ± SEM. *n* = 7–10 mice/genotype/time point; Genotype: F (1, 30) = 10.43; *P < 0.05, 2-way ANOVA followed by Bonferroni’s *t* test. (**C**) Representative movement traces of animals in the OFT. (**D**) The discrimination index of *Cry1Δ11* mice was significantly lower than that of WT controls in the short-term test. Data are presented as mean ± SEM. *n* = 7–10 mice/genotype/time point; Genotype: F (1, 30) = 40.79; ****P < 0.0001, 2-way ANOVA followed by Bonferroni’s *t* test. (**E**) The discrimination index of *Cry1Δ11* mice was significantly lower than that of WT controls in the long-term test during ZT14–16. Data are presented as mean ± SEM. *n* = 7–10 mice/genotype/time point; Genotype: F (1, 30) = 6.741; ***P < 0.001, 2-way ANOVA followed by Bonferroni’s *t* test. (**F**) Spontaneous alternation in the Y-maze test was significantly lower in *Cry1Δ11* mice than that of WT controls. Data are presented as mean ± SEM. *n* = 7–10 mice/genotype/time point; Genotype: F (1, 30) = 28.17; ****P < 0.0001, 2-way ANOVA followed by Bonferroni’s *t* test.

An open field test (OFT) was used to measure spontaneous locomotor activity. During both ZT02–04 and ZT14–16 periods, *Cry1Δ11* mice travelled significantly longer distances and crossed significantly more zones than WT mice (Figure 2, A–C). The percentage of distance traveled in the center zone was also significantly higher for *Cry1Δ11* mice than WT controls (Supplemental Figure 2A), implying a reduced anxiety-like behavior.

ADHD is frequently accompanied by learning difficulty. We therefore performed 2 behaviors related to learning and memory, i.e., novel object recognition (NOR) and Y-maze. As these behavioral tests require that the animals carefully observe their environment, a deficit in learning and memory demonstrated by these tests may also imply inattention (17–19).

In the NOR assay, *Cry1Δ11* mice were not able to discriminate the novel object as well as WT mice at 1 hour after training regardless of whether the assays were performed at ZT14–16 or ZT02–04 (Figure 2D and Supplemental Figure 2, B and C). The discrimination index of *Cry1Δ11* mice was still significantly lower than that of WT mice at 24 hours after training when the assays were performed at ZT14–16; however, no genotypic difference was observed when the assays were performed at ZT02–04 (Figure 2E and Supplemental Figure 2, D and E).

In the Y-maze assay, the number of total arm entries of *Cry1Δ11* mice was significantly higher than that of WT mice (Supplemental Figure 2F), further supporting the hyperactivity of *Cry1Δ11* mice. Furthermore, *Cry1Δ11* mice showed significantly lower alteration than WT mice when the assays were performed at ZT14–16; however, no genotypic difference was observed when the assays were performed at ZT02–04 (Figure 2F). Taken together, *Cry1Δ11* mice exhibited deficits in learning and memory in a circadian time-dependent manner (Table 1).

### Cry1Δ11 mice exhibited impulsivity.

**Figure 3:**
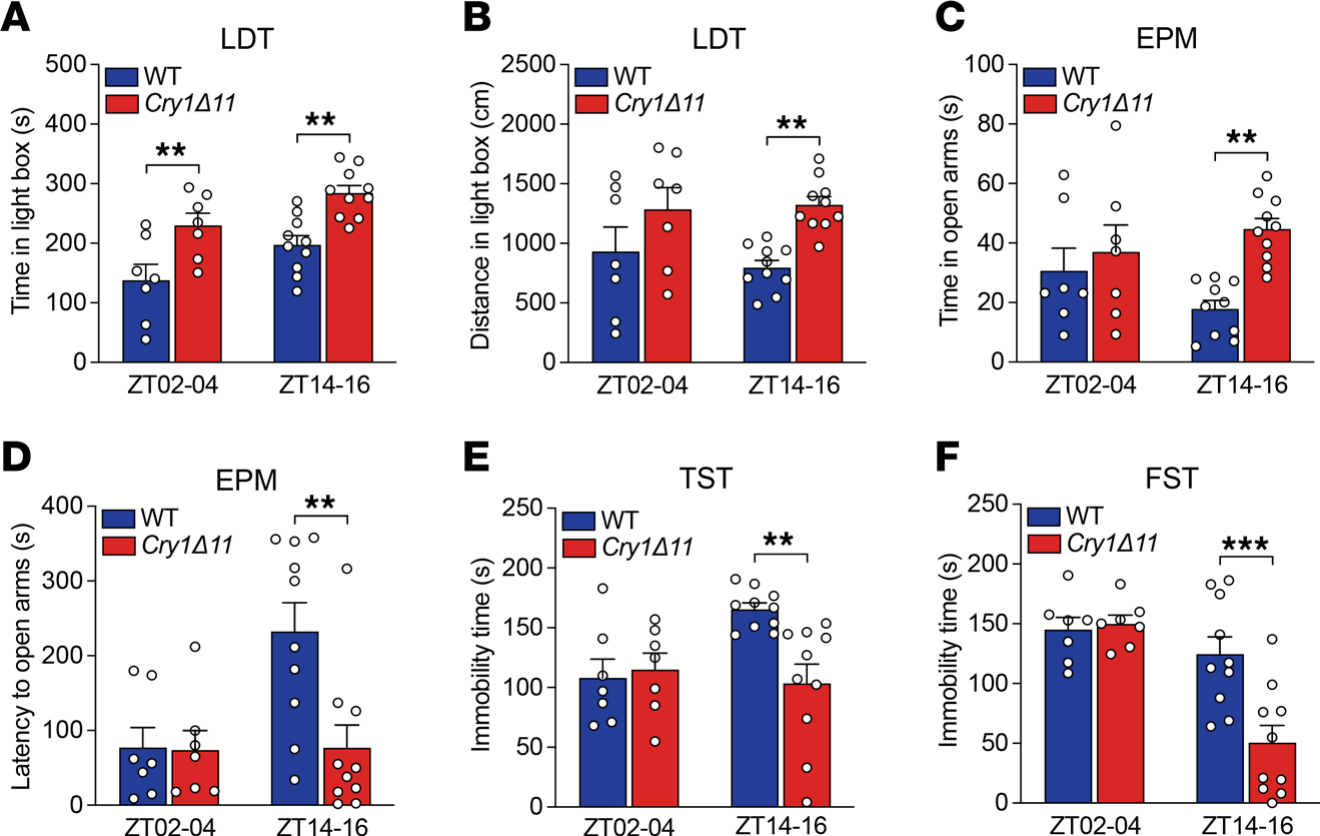
*Cry1Δ11* mice exhibited impulsivity. (**A**) *Cry1Δ11* mice spent significantly more time in the lit box in the LDT test. Data are presented as mean ± SEM. *n* = 7–10 mice/genotype/time point; Genotype: F (1, 30) = 23.81; **P < 0.01, 2-way ANOVA followed by Bonferroni’s *t* test. (**B**) *Cry1Δ11* mice travelled significantly longer distances in the lit box in a LDT measured during ZT14–16. Data are presented as mean ± SEM. *n* = 7–10 mice/genotype/time point; Genotype: F (1, 30) = 12.32; **P < 0.01, 2-way ANOVA followed by Bonferroni’s *t* test. (**C**) *Cry1Δ11* mice spent significantly more time in the open arms in the EPM test measured during ZT14–16. Data are presented as mean ± SEM. *n* = 7–10 mice/genotype/time point; Genotype: F (1, 30) = 8.778; **P < 0.01, 2-way ANOVA followed by Bonferroni’s *t* test. (**D**) Latency to the open arms was significantly shorter in *Cry1Δ11* mice in the EPM measured during ZT14–16. Data are presented as mean ± SEM. *n* = 7–10 mice/genotype/time point; Genotype: F (1, 30) = 5.645; **P < 0.01, 2-way ANOVA followed by Bonferroni’s *t* test. (**E**) Immobility time was significantly shorter in *Cry1Δ11* mice in the TST measured during ZT14–16. Data are presented as mean ± SEM. *n* = 7–10 mice/genotype/time point; Genotype: F (1, 30) = 4.246; **P < 0.01, 2-way ANOVA followed by Bonferroni’s *t* test. (**F**) Immobility time was significantly shorter in *Cry1Δ11* mice in the FST measured during ZT14–16. Data are presented as mean ± SEM. *n* = 7–10 mice/genotype/time point; Genotype: F (1, 30) = 28.17; ***P < 0.001, 2-way ANOVA followed by Bonferroni’s *t* test.

Light-dark transition (LDT) and elevated plus maze (EPM), 2 commonly used assays for anxiety-like behaviors, have also been used to evaluate impulsivity. As shown in Figure 3, A and B, *Cry1Δ11* mice spent significantly more time and travelled significantly longer distances in the lit box in LDT tests measured during ZT14–16. However, when the LDT was performed during ZT02–04, only time spent in the lit box was significantly different between genotypes (Figure 3, A and B). In the EPM assays performed during ZT14–16, *Cry1Δ11* mice spent significantly more time in the open arms (Figure 3C), and the latency to the open arms was also significantly shorter in *Cry1Δ11* mice (Figure 3D). Consistent with the hyperactivity in the OFT, *Cry1Δ11* mice travelled significantly longer distances than WT mice in the EPM test (Supplemental Figure 2G).

We further analyzed depression-like or stress-coping behaviors by using the tail suspension test (TST) and forced swim test (FST). In both the TST and FST, *Cry1Δ11* mice were significantly less despairing, as reflected in the immobile time, than WT mice during ZT14–16, but no genotypic difference was observed during ZT02–04 (Figure 3, E and F). Taken together, *Cry1Δ11* mice showed hyperactivity and reduced anxiety-like and depression-like behaviors in a circadian time-dependent manner (Table 1).

### Hyperactive cAMP signaling in the NAc of Cry1Δ11 mice.

**Figure 4:**
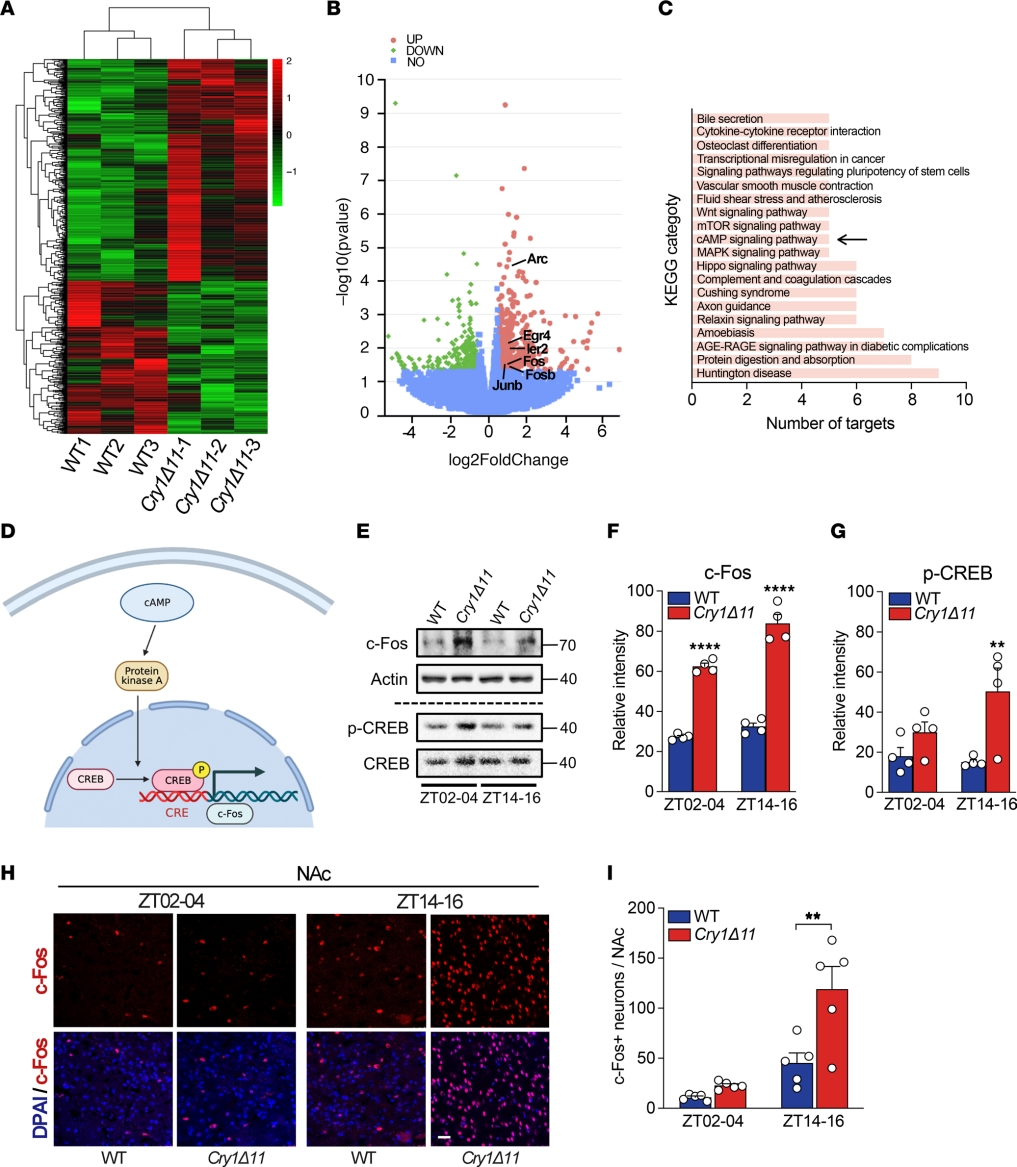
Hyperactive cAMP signaling in the NAc of *Cry1Δ11* mice. (**A**) Gene expression in the NAc of WT and *Cry1Δ11* mice as assayed by RNA-Seq. (**B**) Volcano plot depicting the relative mRNA abundance in the NAc of *Cry1Δ11* mice compared with that of WT controls. Upregulated immediate early genes are indicated. (**C**) KEGG pathway analysis on differentially expressed genes. The cAMP signaling pathway is indicated with an arrow. (**D**) Schematic diagram showing the cAMP signaling pathway. (**E**–**G**) Representative IBs and quantification of lysates from the NAc of WT and *Cry1Δ11* mice taken during ZT02–04 or ZT14–16. c-Fos and p-CREB levels were significantly increased in the NAc of *Cry1Δ11* mice. Data are presented as mean ± SEM. *n* = 4 mice/genotype/time point. (**F**) Genotype: F (1, 12) = 290.1; (**G**) Genotype: F (1, 12) = 12.27; ****P < 0.0001, **P < 0.01, 2-way ANOVA followed by Bonferroni’s *t* test. (**H** and **I**) Representative immunofluorescence images and quantification of c-Fos–positive neurons in the NAc of WT and *Cry1Δ11* mice taken during ZT02–04 or ZT14–16. Scale bar: 10 μm. Data are presented as mean ± SEM. *n* = 4 mice/genotype/time point; Genotype F (1, 16) = 11.80; **P < 0.01, 2-way ANOVA followed by Bonferroni’s *t* test.

To assess the molecular basis underlying the behavioral deficits in *Cry1Δ11* mice, we compared the gene expression profile in the NAc, one of the nuclei relevant to ADHD-like behaviors (20), from *Cry1Δ11* mice and WT controls at ZT14. Using RNA-Seq (Figure 4A), we identified 372 upregulated and 256 downregulated genes in the NAc from *Cry1Δ11* mice (Figure 4, A and B). Interestingly, a panel of immediate early genes, including *Arc*, *Egr4*, *Fos*, *Fosb*, *Ier2*, and *Junb*, were among the significantly upregulated genes (Figure 4B and Supplemental Figure 3). The Kyoto Encyclopedia of Genes and Genome (KEGG) analysis revealed enrichment of the differentially expressed genes in several pathways, notably the cAMP signaling pathway (Figure 4C).

We then measured levels of phosphorylated cAMP response element–binding protein (p-CREB) and c-Fos to verify that the cAMP signaling pathway had been altered (Figure 4D). As shown in Figure 4, E–G, both p-CREB and c-Fos were significantly upregulated in the NAc taken from *Cry1Δ11* mice as compared with their WT controls, regardless of whether the samples were taken during ZT2–4 or ZT14–16. Immunofluorescence analysis revealed an increase in the number of c-Fos–positive neurons in the NAc, but not in the PFC or hippocampus, from *Cry1Δ11* mice (Figure 4, H and I, and Supplemental Figure 4). Taken together, our results uncovered a hyperactive cAMP signaling pathway in the NAc of *Cry1Δ11* mice.

### Hyperactive DRD1 signaling in Cry1Δ11 mice.

**Figure 5:**
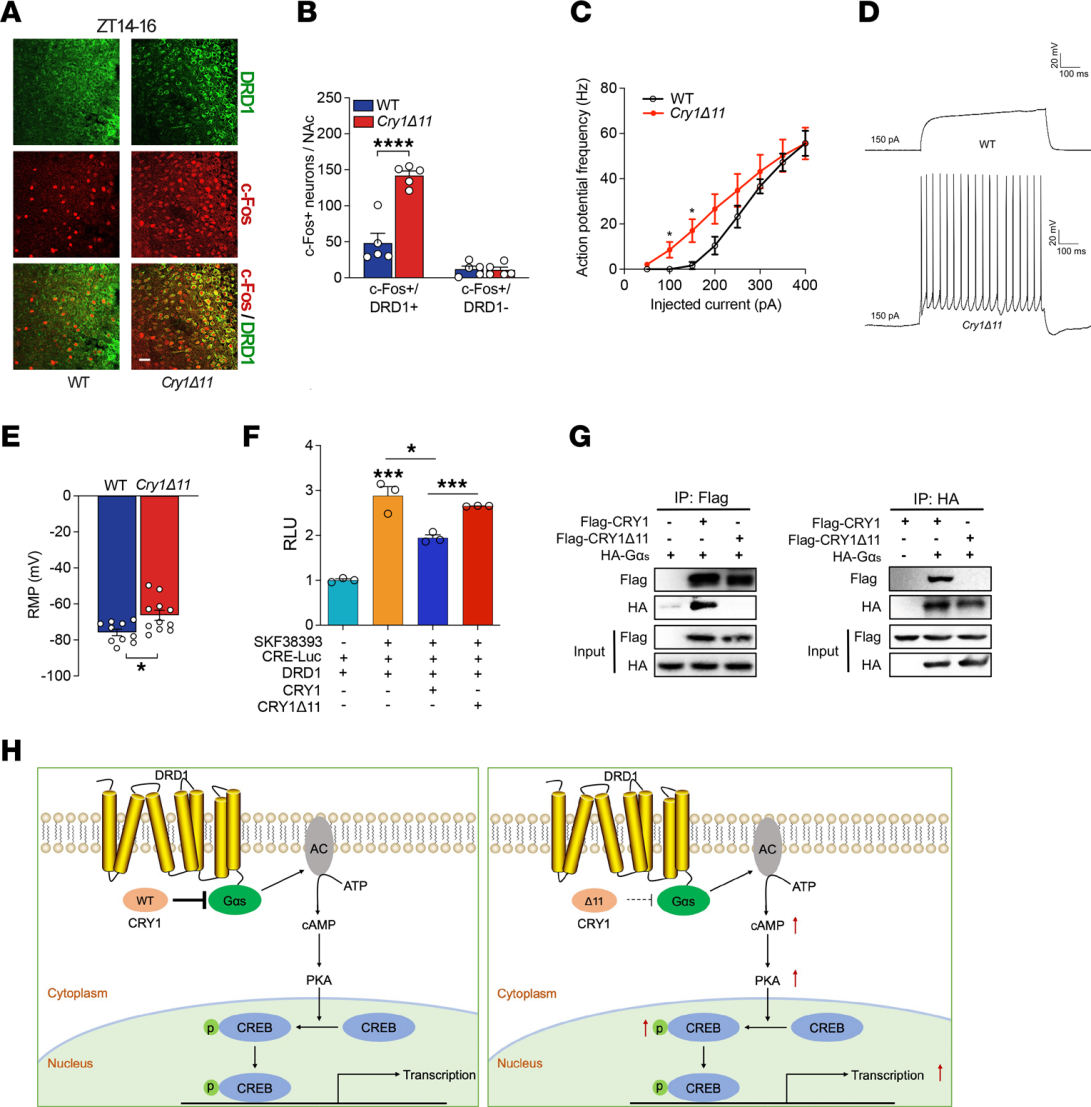
Hyperactive DRD1 signaling in *Cry1Δ11* mice. (**A** and **B**) Representative immunofluorescence images and quantification of neurons expressing both c-Fos and DRD1 in the NAc of WT and *Cry1Δ11* mice taken during ZT14–16. Scale bar: 10 μm. Data are presented as mean ± SEM. *n* = 5 mice/genotype, Genotype F (1, 16) = 33.40; ****P < 0.0001, 2-way ANOVA followed by Bonferroni’s *t* test. (**C**) The frequency of action potentials of DRD1-MSNs in the NAc induced by 400-pA current steps was significantly higher in *Cry1Δ11* mice. Data are presented as mean ± SEM; *n* = 10–11 mice/genotype; *P < 0.05, repeated-measure ANOVA test. (**D**) Representative action potentials of DRD1-MSNs in NAc induced by 150-pA current steps. (**E**) The resting membrane potential of DRD1-MSNs in the NAc taken from *Cry1Δ11* mice was significantly more depolarized than that of WT mice. Data are presented as mean ± SEM. *n* = 10 neurons from 10 WT mice, and *n* = 11 neurons from 10 *Cry1Δ11* mice. *P < 0.05, unpaired 2-tailed Student’s *t* test. (**F**) *Cry1Δ11* failed to inhibit DRD1-signaling, in contrast to the inhibition produced by WT *Cry1*, as demonstrated by a luciferase assay. Data are presented as mean ± SEM; *n* = 3; ***P < 0.001, unpaired 2-tailed Student’s *t* test. RLU, relative luminescence units. (**G**) In contrast to WT *Cry1*, *Cry1Δ11* lost the ability to interact with Gαs, as assayed by a co-IP assay. Abs against HA or FLAG were used to IP cell lysates, and the immune complexes were blotted with Ab against HA or FLAG, respectively. (**H**) Diagram depicting the mechanism underlying hyperactive DRD1 signaling induced by the *Cry1Δ11* mutation.

The NAc contains 2 functionally distinct types of neurons: medium spiny nerve cells (MSNs) expressing either the DRD1 or the DRD2. We therefore used colocalization immunofluorescence to analyze the distribution of c-Fos in the DRD1-positive and -negative MSNs, respectively. As shown in Figure 5, A and B, most of the c-Fos was expressed in DRD1^+^ MSNs; the c-Fos^+^/DRD1^+^ neurons were significantly increased in the NAc of *Cry1Δ11* mice, whereas there was no genotypic difference in the number of c-Fos^+^/DRD1^–^ neurons.

To further confirm the hyperactivity in DRD1-MSNs, we performed whole-cell patch clamp recordings in these neurons. Neurons that showed higher action potential (AP) firing frequency (see Methods) after the treatment of DRD1 agonist SKF38393 were identified as putative DRD1^+^ MSNs; otherwise, they were defined as DRD1^–^ MSNs (Supplemental Figure 5, A and D). As shown in Figure 5, C–E, the putative DRD1^+^ MSNs from *Cry1Δ11* mice exhibited a significantly higher AP frequency and depolarizing resting membrane potential (RMP) than those from the WT controls. Further analysis in putative DRD1^+^ MSNs also revealed a decrease of rheobase from *Cry1Δ11* mice (Supplemental Figure 5B), indicating a hyperexcitability of these DRD1^+^ MSNs. This hyperactivity is not attributed to the change of cell input resistance (Rin) (Supplemental Figure 5C). Nevertheless, no genotypic difference was found in DRD1^–^ MSNs (Supplemental Figure 5E). Taken together, our data indicate that the DRD1 signaling pathway in *Cry1Δ11* mice was hyperactive.

### Cry1Δ11 lost the ability to inhibit Gαs.

In a seminal study, Zhang et al. demonstrated that CRY1/2 proteins are able to suppress cAMP signaling, presumably through interaction with Gαs (21). DRD1 is a receptor coupled to Gαs, and CRY1/2 indeed suppressed DRD1 signaling (21, 22). We applied a luciferase reporter assay to see if deletion of exon 11 influences the inhibitory effect of Cry1 on DRD1 signaling. As shown in Figure 5F, DRD1 agonist SKF38393 induced a significant increase in luciferase activity when DRD1 and CRE-luciferase were coexpressed in HEK293 cells. Overexpression of WT Cry1, but not Cry1Δ11, significantly suppressed the SKF38393-induced luciferase activity. We further used co-IP assays to see if deletion of exon 11 affected the interaction between Cry1 and Gαs proteins. As shown in Figure 5G, only when HA-tagged Gαs and FLAG-tagged CRY1 were coexpressed in HEK 293T cells, was the FLAG Ab able to pull down HA-tagged Gαs at the same time that the HA Ab was able to pull down FLAG-tagged CRY1, indicating the existence of a specific interaction between Cry1 and Gαs proteins. However, such an interaction was absent between Cry1Δ11 and Gαs proteins. We thus propose a working model: Cry1Δ11 fails to interact and inhibit Gαs as can its WT control, leading to hyperactive DRD1 signaling in *Cry1Δ11* mice (Figure 5H).

### A DRD1 antagonist rescued the ADHD-like behaviors in Cry1Δ11 mice.

**Figure 6:**
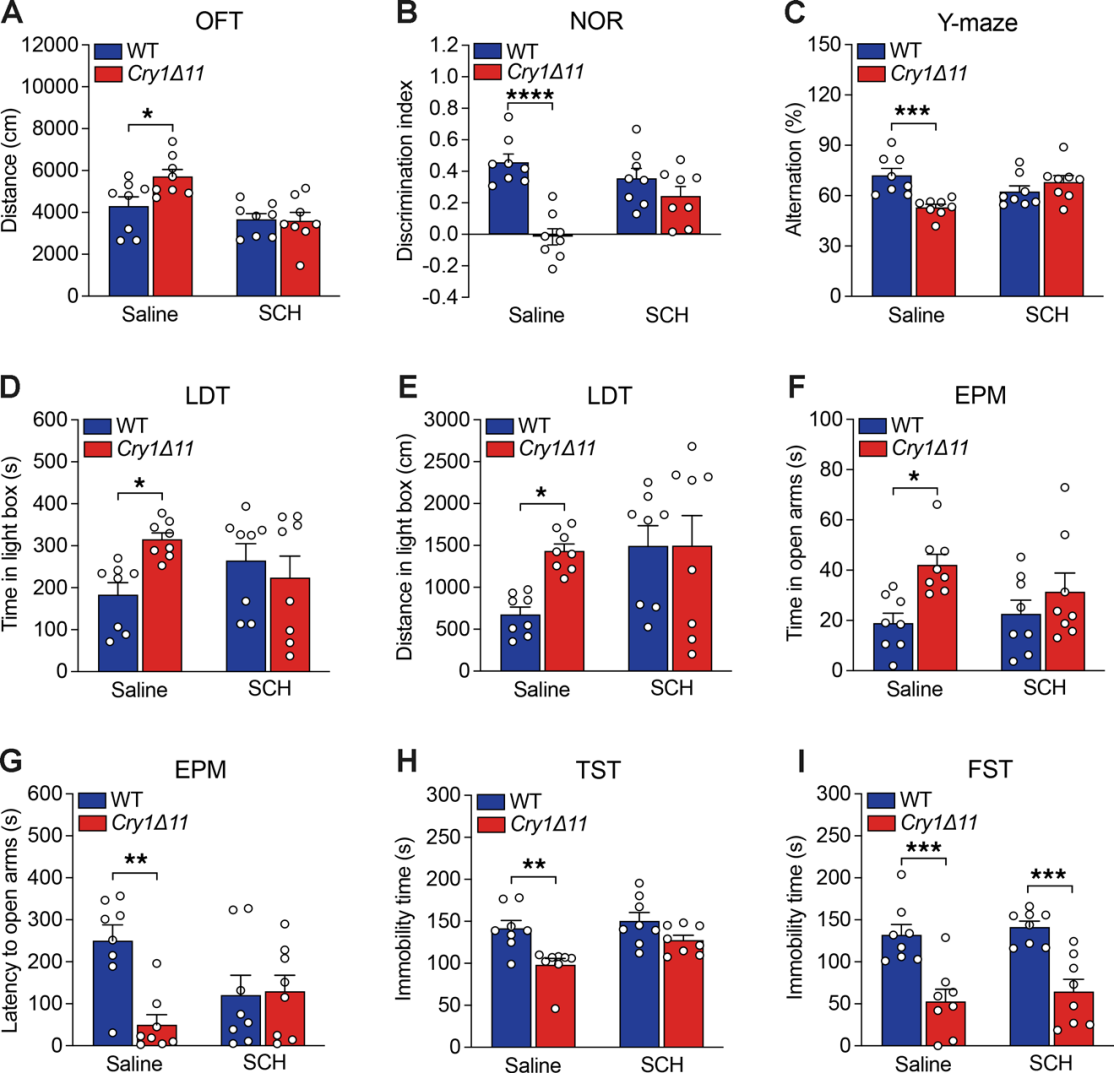
Inhibition of DRD1 signaling rescued the ADHD-like behaviors in *Cry1Δ11* mice. Saline or DRD1 antagonist SCH23390 (15 μg/kg) were injected i.p. during ZT14–16, and behaviors were measured at 30 minutes after injection. SCH23390 normalized the hyperactivity in the OFT test, (**A**) rescue learning and memory deficits in the NOR (**B**) and Y-maze (**C**) tests, reduced anxiety-like behaviors in the LDT (**D** and **E**) and EPM tests (**F** and **G**), reduced depression-like behaviors in the TST test (**H**), but had no effect on the reduced depression-like behaviors in the FST test (**I**). Data are presented as mean ± SEM; *n* = 8 mice/genotype/treatment; Genotype: F (1, 28) = 3.265 (**A**), 26.57 (**B**), 3.887 (**C**), 1.558 (**D**), 2.843 (**E**), 8.683 (**F**), 6.502 (**G**), 15.95 (**H**), 38.98 (**I**); *P < 0.05, **P < 0.01, ***P < 0.001, ****P < 0.0001, 2-way ANOVA followed by Bonferroni’s *t* test.

To investigate whether hyperactive DRD1 signaling is responsible for the ADHD-like behaviors in *Cry1Δ11* mice, we treated mice with the DRD1 antagonist SCH23390 (15 μg/kg, i.p.) and measured their behaviors 30 minutes later. All experiments were carried out during ZT14–16 when ADHD-like symptoms were consistently observed in *Cry1Δ11* mice. Except for the immobile time in the FST assay, the hyperactivity, impaired learning and memory, reduced anxiety-like behaviors, and the reduced immobile time in the TST assay were normalized by SCH23390 (Figure 6, A–I). Taken together, these results suggest that DRD1 antagonists may be a potential therapeutic approach for ADHD induced by the *Cry1Δ11* mutation (Table 1).

## Discussion

In this study, we demonstrated that *Cry1Δ11* mice showed ADHD-like symptoms. We further identified that hyperactive DRD1 signaling may be the underlying pathological mechanism. Importantly, the DRD1 antagonist SCH23390 normalized most ADHD-like behaviors in *Cry1Δ11* mice, supporting DRD1 inhibition as a potential therapeutic target for ADHD.

### Circadian phenotype in Cry1Δ11 mice.

*CRY1Δ11* was initially identified in individuals with DSPD (15); however, we did not observe significant differences in the free-running period in *Cry1Δ11* mice. By using a bioluminescence assay, Patke et al. demonstrated that expression of human CRY1Δ11 in *Cry1/2* double-deficient mouse embryonic fibroblasts (DKO MEFs) increased the circadian period by approximately half an hour as compared with expression of full-length human CRY1. It should be noted that only full-length or Δ11 form of CRY1 was expressed in the DKO MEFs. However, the Δ11 form of the Cry1 protein was coexpressed with 1 copy of full-length Cry1 and 2 copies of full-length Cry2 in the *Cry1Δ11* mice, equivalent to the status of patients with DSPD carrying heterozygous CRY1 c. 1657 + 3A > C. Further studies under different conditions, such as constant lighting, may be required to understand the mechanisms underlying the DSPD phenotype in humans with CRY1Δ11 variants.

### Cry1Δ11 mice versus Cry1-KO mice.

In this study, we demonstrated that *Cry1Δ11* mice showed reduced anxiety-like and depression-like behaviors. In contrast, *Cry1*-KO mice were reported to show an increase or no change in depression-like behaviors in different studies, although reduced anxiety-like behaviors were also reported in these mice (23). First, the time of day when the behaviors were performed may confound the data, as we have shown in this study that *Cry1Δ11* mice showed stronger behavioral phenotypes during ZT14–16. On the other hand, it should be noted that Cry1 may affect the dopamine system through several different pathways. In addition to suppressing DRD1 signaling through inhibiting Gαs protein, Cry1 is also able to inhibit the expression of circadian clock-related genes, such as *Rev-erba* and *Per2* (6), which in turn influence dopamine synthesis/degradation (24, 25). As such, KO of *Cry1* per se may lead to a complicated effect on the dopamine system. However, Cry1Δ11 showed an enhanced inhibitory effect on BMAL1:CLOCK heterodimer but lost the inhibitory effect on DRD1 signaling (15). Therefore, *Cry1* KO and the *Cry1Δ11* mutation seem to have distinct effects on the dopamine system, which can partially explain the discrepancy between *Cry1*-KO and *Cry1Δ11* mice. Interestingly, Porcu et al. demonstrated that DRD1-MSN–specific knockdown of *Cry1/2* in the NAc reduced susceptibility to stress-induced helplessness, similar to the reduced depression-like behaviors in *Cry1Δ11* mice (22). In this scenario, it seems that only DRD1 signaling in the NAc is affected. Therefore, *Cry1Δ11* instead of *Cry1*-KO mice provided an excellent opportunity to study the contribution of DRD1 signaling to neuropsychiatric disorders such as ADHD.

### Circadian clock genes and the dopamine system.

Dopamine synthesis and secretion follow a diurnal cycle that is in close interaction with the circadian rhythms of several clock genes and proteins. Intriguingly, locomotor hyperactivity and impaired dopaminergic signaling are commonly reported in mice lacking circadian clock genes. For instance, *ClockΔ19* mutant mice have increased dopamine excitability in the neurons of the ventral tegmental area and are more sensitive to the rewarding effects of cocaine (26).

Circadian clock proteins may influence dopaminergic signaling in different ways, such as by dopamine synthesis, degradation, and signal transduction. Rev-erbα is reported to suppress the transcription of tyrosine hydroxylase, the rate-limiting enzyme in dopamine production (24). In consequence, *Rev-erba*–KO mice show an increase in dopamine turnover, which accounts for their locomotor hyperactivity and reduced anxiety and depression-like behaviors.

The promoter of monoamine oxidase A (MaoA), an enzyme that is involved in dopamine catabolism, is regulated by the clock components BMAL1, NPAS2, and PER2. Therefore, a deficiency in *Per2* results in increased levels of dopamine, which may account for reduced depression-like behaviors in *Per2*-KO mice (25). Zebrafish lacking the *PER1b* gene (an analog of the human *PER1* gene) and *Per1*-KO mice display hyperactive, impulsive-like, and cognitive deficit-like behaviors (12). Mechanistically, transcription of *Mao* and *Dbh*, 2 genes involved in DA catabolism, are activated by the CLOCK:BMAL1 heterodimer but are repressed by Per1b. Thus, loss of *Per1b* leads to upregulation of *Mao* and *Dbh*, which may in turn result in dopamine reduction and, thus, contribute to ADHD-like symptoms (12).

Here, we demonstrated that Cry1, but not Cry1Δ11, suppressed DRD1 signaling by interacting with and inhibiting Gαs protein. Therefore, *Cry1Δ11* mice showed hyperactive DRD1 signaling, which may underlie their ADHD-like symptoms. Therefore, circadian clock genes may impinge in various nodes of the dopamine system to affect mood, which may underlie psychiatry disorders induced by disrupted circadian rhythms.

### Circadian variation in mood-related behaviors.

Diurnal variation in mood is a prominent symptom for psychiatric disorders. For instance, depression shows a tendency toward maximal symptom severity in the morning (27). In this study, we demonstrated that the ADHD-like behaviors in *Cry1Δ11* mice are more severe at dusk than at dawn, which is consistent with the higher expression of Cry1 in several brain areas at dusk. Interestingly, the phenotypes in *Rev-erbɑ*–KO mice are also more severe in the dusk, when its expression in the brain is relative higher (24). However, mice with dysfunctional Bmal1 in NAc astrocytes show obvious phenotypes during the light phase, including increase in locomotor response to novelty, exploratory drive, operant food self-administration, and motivation (28). Moreover, McClung and colleagues studied the cocaine self-administration behaviors in WT and *ClockΔ19 mice* at dusk (ZT2) and at dawn (ZT14), respectively (26). They demonstrated that only *ClockΔ19* mice acquired cocaine self-administration at ZT2. It is not clear if the circadian rhythms of BMAL1/CLOCK expression in specific brain regions underlie the diurnal variation in these behaviors. However, considering the diurnal variation in the expression of a vast number of genes, we strongly suggest analyzing the phenotypes (especially behaviors) of gene KO/mutant animals at different circadian time points according to their expression patterns.

### Limitation and perspective.

ADHD is a disorder with several symptoms, including hyperactivity, inattention, and impulsivity. Even some ADHD patients only exhibit some, but not all symptoms (2). Thus, it is not always easy to characterize the ADHD-related behaviors in rodents. Although we compared a panel of ADHD-relevant behaviors in *Cry1Δ11* mice and their WT controls, more comprehensive assays, such as 5-choice serial reaction time task (29), if available, should be applied to further validate the ADHD-like symptoms in *Cry1Δ11* mice. Furthermore, although we showed that hyperactive DRD1 signaling seems to be responsible for the ADHD-like symptoms in *Cry1Δ11* mice, specific brain areas involved in these symptoms are still unknown. Pharmacological or genetic manipulation in specific neurons may be able to dissect the underlying neural circuits.

## Methods

### Animals.

*Cry1Δ11* mice were generated using CRISPR/Cas9 technology. Donor DNA of 96 bp in length, sgRNA, and in vitro–transcribed Cas9 mRNA were microinjected into E0.5 zygotes. Sequences for donor DNA and sgRNA are shown in Figure 1B. The injected zygotes were transplanted into foster mother mice. DNA samples from tail biopsies of offspring were amplified by PCR using the following primers: forward, GCAAGAGATGGGAAGACCAG, and reverse: TACTAAAGCAGGGAAGAGAAGACC. The PCR products were Sanger sequenced to identify the mutation. Mice of the same sex were group housed (3–5 animals per cage) under controlled conditions (temperature, 20°C ± 2°C; relative humidity, 50%–60%; 12-hour LD cycle, lights on at 7 am and lights off at 7 pm) and had free access to food and water. All procedures regarding the care and use of animals were approved by the IACUC of Central South University of China.

### Circadian behaviors.

Male *Cry1Δ11* or WT mice at the age of 4–6 months were individually housed within cages equipped with running wheels and were allowed free access to food and water. Their locomotor activities were recorded as revolutions per 5-minute intervals. Mice were entrained to an initial LD cycle (light intensity ~150 lx, lights on at 7 am and lights off at 7 pm). After 2–3 weeks of recording activity under LD conditions, the mice were placed in DD for 3–4 weeks. The free-run period was calculated using ClockLab software (Actimetrics) in the MATLAB environment (MathWorks).

### Behavioral tests.

Male *Cry1Δ11* and their littermate WT controls (*n* = 7–10) at the age of 8–12 weeks were used for behavioral tests. Four batches of mice were used in this study (Supplemental Table 1). Each mouse was tested according to the following order: OFT—NOR—Y-maze—LDT—EPM—TST—FST. Mice were allowed to rest for 1–2 days after each behavioral test.

### OFT.

Locomotion activity was measured by OFT. On test day, male *Cry1Δ11* or WT mice with littermate controls were transported to the testing room and left undisturbed for 1 hour before testing. Each animal was placed in the center of the arena (72 × 72 × 36 cm) and video recorded for 10 minutes. Total distance traveled and number of central zone (36 × 36 cm) crossings were calculated.

### LDT.

The LDT utilizes the natural aversion of rodents to bright light and open field; thus, animals with reduced anxiety will spend relatively more time in the lit compartment. The apparatus was divided into a lit compartment (30 × 20 × 25 cm, ~300 lux) and a dark compartment (20 × 15 × 25 cm) connected by a 4 cm^2^ tunnel. Male *Cry1Δ11* or WT mice with littermate controls were initially placed in the dark compartment. Time spent in the lit compartment and distance travelled in the lit compartment were video recorded and quantified over a 10-minute period.

### EPM.

The plus maze consisted of 2 open (30 × 5 cm) and 2 wall-enclosed arms (30 × 5 × 15 cm) connected by a central platform (5 × 5 cm). The apparatus was elevated 50 cm above the floor. Behavioral testing began by placing a mouse in the central area facing a closed arm. Exploratory behavior was monitored over a period of 8 minutes. Time traveled in open and closed arms and latency until the first open-arm entry were recorded and quantified. Entries were defined as 4 paws of an animal entering a new zone.

### Depression-like behaviors.

The TST and Porsolt’s FST were used to assess depression-like behaviors. For the TST, male *Cry1Δ11* or WT mice with littermate controls were individually suspended by the tail in a white box (40 × 20 × 8 cm) for 6 minutes. They were considered immobile when agitation and escape attempts ceased. For the FST, mice were placed in a transparent beaker (18.5 cm diameter) containing water (21°C–25°C) at a depth of 15 cm for 6 minutes. Immobility was defined as the absence of volitional body or limb movement. The time spent immobile during the last 4 minutes was quantified.

### NOR.

Male *Cry1Δ11* and their littermate WT mice were placed and habituated in a square chamber (33 × 33 × 20 cm) with 2 identical objects for 10 minutes. At 1 hour or 24 hours after habituation, 1 object was replaced with a novel object and the mouse was placed back into the chamber. The behavior of the mouse as it explored objects in its vicinity was video recorded for 10 minutes and quantified. The data were recorded once the mice were touching the objects. The time spent on exploring the familiar (F) object, new (N) object, and whole time (N + F) were recorded to estimate the discrimination index = (N – F)/(N + F).

### Y-maze.

The Y-maze spontaneous alternation paradigm is based on the natural tendency of rodents to explore a novel environment. The apparatus consisted of 3 opaque arms (30 × 5 × 15 cm) that radiate from the center in a Y shape. The behavioral test was initiated by placing the mouse in the center of the Y, allowing free access to any of the 3 arms. The movement of the mouse was tracked by a video camera for 8 minutes and quantified. An arm entry was counted when all 4 paws of the mouse entered the arm; a “spontaneous alternation” is defined as a set of choices of consecutive arms without a repeated entry.

### Drug administration.

SCH23390 (15 μg/kg; Selleck, S0476) and SKF38393 were dissolved in saline and injected i.p.

### RNA-Seq.

Brains were extracted and flash frozen from 8 weeks for male *Cry1Δ11* or WT mice. The NAc tissue was collected at ZT14. Individual mice were used as biological replicates (3 mice/genotype). Total amounts and integrity of RNA were assessed using the RNA Nano 6000 Assay Kit of the Bioanalyzer 2100 system (Agilent Technologies). The library fragments were purified with AMPure XP system (Beckman Coulter). The mRNA was purified from total RNA by using poly-T oligonucleotide–attached magnetic beads. The library fragments were purified with AMPure XP system (Beckman Coulter). After PCR amplification, the PCR product was purified by AMPure XP beads, and the library was finally obtained. The library was initially quantified by Qubit 2.0 fluorometer and then sequenced by the Illumina NovaSeq 6000. The image data measured by the high-throughput sequencer were converted into sequence data (reads) by CASAVA base recognition. The index of the reference genome was built using Hisat2 (v2.0.5) and featureCounts (v1.5.0-p3) was used to count the read numbers mapped to each gene. Differential expression analysis of 2 groups was performed using the DESeq2 R package (1.20.0). The resulting *P* values were adjusted using the Benjamini and Hochberg’s approach for controlling the FDR. Padj of 0.05 or less and |log_2_ (foldchange)| of 1 or greater were set as the threshold for significantly differential expression. We used clusterProfiler R package (v3.8.1) to test the statistical enrichment of differential expression genes in KEGG pathways. Reactome pathways with corrected *P* values less than 0.05 were considered significantly enriched by differential expressed genes. All original RNA-Seq data were deposited in the NCBI, under the Bioproject accession PRJNA990506.

### Cell culture.

HEK293T cells were maintained in DMEM supplemented with 10% FBS, 100 U/mL penicillin, and 100 μg/mL streptomycin at 37°C in 5% CO_2_ incubators. Transfections were performed with Lipofectamine 2000 (Invitrogen) reagents according to the manufacturer’s protocol.

### Luciferase reporter assay.

The CRE-luciferase plasmid (Promega, E847A) and Renilla-luciferase plasmid (Promega, E2231) were transfected with the indicated plasmids into HEK293 cells. At 2 days after transfection, cells were serum starved for 12 hours and treated with the DRD1 agonist SKF38393 (1 μM; Selleck, S7993) for 4 hours. Afterward, the cells were lysed with luciferase lysis buffer and the luminescence levels of firefly luciferase and renilla luciferase were measured with a Berthold Sirius luminometer (Berthold).

### Co-IP.

Cells were harvested with lysis buffer (150 mM NaCl, 1% NP-40, 2 mM EDTA, 50 mM Tris, pH 8.0, and protease inhibitor cocktail). Approximately 1 mg of whole cell lysate was incubated with 1 μg of the indicated Ab with constant agitation overnight at 4°C. Then, 30 μL protein G agarose bead slurry (Sigma-Aldrich, P3296) was added to pull down the immunocomplexes. The beads were collected by centrifugation, washed extensively with lysis buffer 3–5 times, and boiled with 2× SDS loading buffer. The protein samples were then subjected to immunoblot analysis with the appropriate Ab.

### Immunofluorescent staining.

Mice were perfused intracardially with 4% paraformaldehyde, and brains were removed, postfixed, and cryoprotected in 25% sucrose overnight. Sections that were 20 μm thick, including the NAc region, were collected and incubated with primary Abs to c-Fos (diluted 1:400; SYSY, P12841), p-CREB (diluted 1:400; CST, 9198), and DRD1 (diluted 1:400; Sigma-Aldrich, D2944) overnight. The slices were then incubated in the dark with Alexa Fluor–conjugated labeled secondary Abs. Nuclei were stained with DAPI. Slices were imaged using a confocal microscope (TCS SP5; Leica). Fluorescence intensities of c-Fos and p-CREB were analyzed using fluorescence image analysis software (Lumina Vision, Mitani).

### RNA isolation, RT.

Tissues were lysed with TRIzol reagent (Thermo Fisher Scientific, 15596026) according to the manufacturer’s instructions. Then, 2 μg of total RNA were reversed transcribed using RevertAid First Strand cDNA Synthesis Kit (Thermo Fisher Scientific, K1622).

### Western blotting.

Cells or tissue samples were lysed in 2× SDS lysis buffer (2% SDS, 63 mM Tris-HCl, and 10% glycerol). Proteins in lysates were separated by SDS-PAGE, transferred to PVDF, and underwent IB with the corresponding Abs overnight at 4°C. Membranes were then washed and incubated with HRP-conjugated secondary Abs. The proteins were visualized using the Pierce ECL Western Blotting Substrate kit (Thermo Fisher Scientific, 32106). Band intensities were quantified by ImageJ (NIH).

### Electrophysiological recording and analysis.

Electrophysiological experiments were performed in coronal brain slices containing NAc. Brain slices (300–350 μM) were prepared from 6- to 8-week-old male mice. The mice were deeply anesthetized by sodium pentobarbital (50 mg/kg, i.p.). Following decapitation, the brains were rapidly immersed in an ice-cold sucrose-based artificial cerebrospinal fluid (ACSF) containing 2.5 mM KCl, 2 mM MgSO_4_, 2 mM CaCl_2_, 26 mM NaHCO_3_, 1.25 mM NaH_2_PO_4_·H_2_O, 10 mM dextrose, 213 mM sucrose, and pH 7.2–7.3. Brain blocks were also sliced in this ice-cold ACSF. After slicing, they were immediately transferred to an incubation chamber filled with normal ACSF (126 mM NaCl, 2.5 mM KCl, 2 mM MgSO_4_, 2 mM CaCl_2_, 26 mM NaHCO_3_, 1.25 mM NaH_2_PO_4_, 25 mM dextrose, pH 7.2–7.3) and maintained at 35.5°C for 45–60 minutes and then at room temperature before use. Slices were perfused with carbogenated normal ACSF at 34°C–35°C, at a rate of 2 mL/min.

For whole-cell recording, patch pipettes with an impedance of 4–7 MΩ were filled with normal internal solution containing (in mM) 140 K-Gluconate, 3 KCl, 2 MgCl_2_·6H_2_O, 0.2 EGTA, 10 HEPES, and 2 Na_2_ATP (285–295 mOsm, pH 7.2–7.25) and possessed an impedance of 4–7 MΩ. Neurons within NAc were visually identified under a microscope equipped with IR-DIC optics (BX-51WI, Olympus). Recordings with series resistance less than 20 MΩ were included for data analysis. Current clamp recordings were achieved using a MultiClamp 700B amplifier (Molecular Devices). Digidata 1550B with pCLAMP 10.6 were used for signal acquisition. Signals were filtered at 10 (voltage clamp) or 2.2 kHz (current clamp) and sampled at 50 kHz.

Positive (50 pA/step, 500 ms in duration) or negative (–60 pA, 500 ms) current pulses were given to examine electrophysiological properties such as F–I curve and input resistance. The cell membrane potential without any current injection was defined as RMP. The liquid junction potential (~15 mV) was not corrected in the data analysis process. A successful AP was defined when the amplitude surpassed 30 mV. The rheobase was defined as the minimum current pulse size when AP initiated. A putative DRD1^+^ MSN was defined by the ΔAP firing frequency comparing from control condition to DRD1 agonist (SKF38393, 10 μM) application when the same current size pulses were injected. The ΔAP frequency should be 10% greater than the frequency in the control condition, and only at least 2 successive increasements made would be involved.

### Statistics.

Statistics were performed using GraphPad Prism 9.0. The 2-tailed Student’s *t* test was used for statistical comparison between 2 groups and 2-way ANOVAs or multiple-comparison tests were utilized with Bonferroni’s post hoc test for 3 or more conditions. A *P* value of less than 0.05 was considered significant.

### Study approval.

All procedures were conducted under a protocol approved by the Ethics Committee, Central South University (DWSY-2021-0507).

## Author contributions

JDL and DFL designed the methodology and conducted the investigation. JDL and DFL completed formal raw data analyses and curated the raw data. ZYX and YCZ gave assistance to supplementary experiments. YS, SXD, ZHC, YCZ, PYG, XYL, and XYW provided insights or reagents. All authors analyzed the processed data. JDL and DFL wrote the original draft, which all other authors reviewed and edited. JDL supervised the project. Funding was acquired by JDL, ZHC, and DFL.

## Supplementary Material

Supplemental data

Supporting data values

## Figures and Tables

**Table 1:**
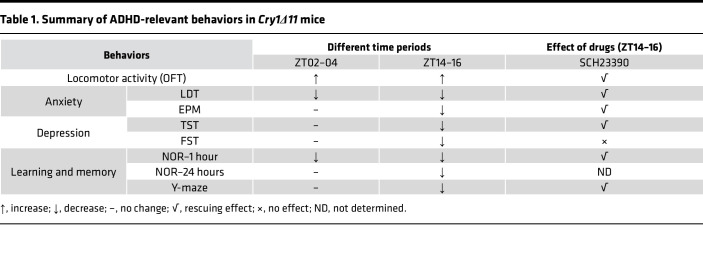
Summary of ADHD-relevant behaviors in *Cry1Δ11* mice

## Version 1 08/22/2023

Electronic publication

## Version 2 08/31/2023

The column headings in Table 1 were corrected
