# Supplementary material for: Cry1Δ11 mutation induces ADHD-like symptoms through hyperactive dopamine D1 receptor signaling
Source: JCI Insight. 2023 Aug 22;8(16):e170434. doi: 10.1172/jci.insight.170434 (PMC10543712; doi:10.1172/jci.insight.170434)
Supplement: Supplemental data [file jciinsight-8-170434-s281.pdf]

Cry1Δ11 mutation induces ADHD-like symptoms through hyperactive dopamine D1 receptor signaling

Dengfeng Liu[1], Zhengyu Xie[2], Panyang Gu[1], Xiangyu Li[1], Yichun Zhang[1], Xinying Wang[1], Zhiheng Chen[3], Suixin Deng[4], Yousheng Shu[1,4], Jia-Da Li[1,5,6,7]

Supplemental data information

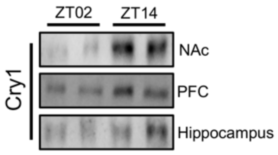

Figure S1 Western blot analysis of Cry1 protein in the NAc, PFC and hippocampus of wild-type C57BL6 mice taken at ZT02 and ZT14, respectively.

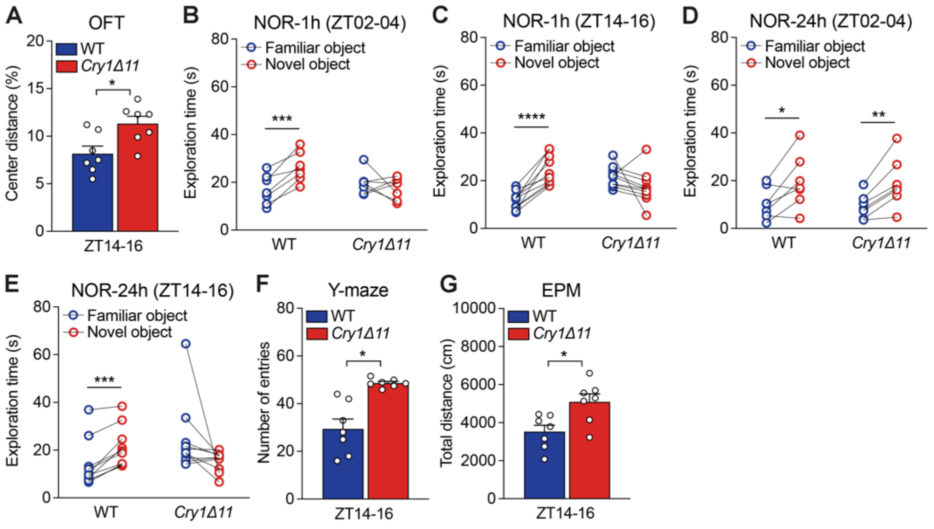

Figure S2 (A) The percentage of distance traveled in the center zone was significantly higher for *Cry1Δ11* mice as assayed in the OFT test. Data are presented as means ± SEM. n=7 mice/genotype, *p < 0.05, unpaired Student's *t* test. (B-C) The exploration time on a novel object and a familiar object of animals in the short term NOR test performed during ZT02-04 (B) or ZT14-16 (C). Data are presented as means ± SEM.

n=7-10 mice/genotype/time point, ***p < 0.001, ****p < 0.0001, paired t test. (D-E) The exploration time on a novel object and a familiar object of animals in the long term NOR test performed during ZT02-04 (D) or ZT14-16 (E). Data are presented as means ± SEM. n=7-10 mice/genotype/time point, *p < 0.05, **p < 0.01, ***p < 0.001, paired t test. (F) *Cry1Δ11* mice travelled significantly longer distances in all arms in the EPM test. Data are presented as means ± SEM. n=7 mice/genotype, *p < 0.05, unpaired Student's *t* test. (G) The total number of entries in the Y-maze test was significantly higher in *Cry1Δ11* mice than WT controls. Data are presented as means ± SEM. n=7 mice/genotype, *p < 0.05, unpaired Student's *t* test.

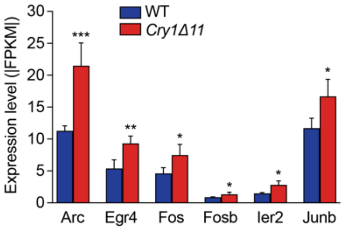

Figure S3 A panel of immediate early genes (IEGs) were significantly elevated in the NAc from *Cry1Δ11* mice. Data are presented as means ± SEM. n=3 mice/genotype, *p < 0.05, **p < 0.01.

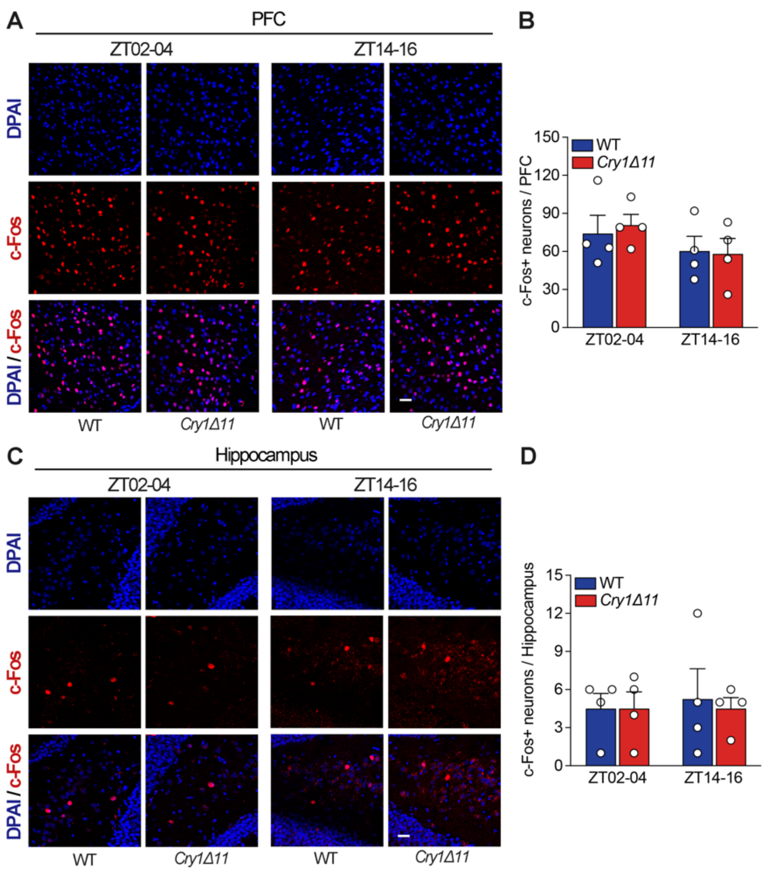

Figure S4 There was no genotypic difference in the number of c-Fos-positive neurons in the PFC (A-B) or hippocampus (C-D). Data are presented as means ± SEM. n=4 mice/genotype/time point, Genotype: $F(1, 30) = 0.03613$ (B), 0.05832 (D), $p > 0.05$, two-way ANOVA followed by Bonferroni *t* test.

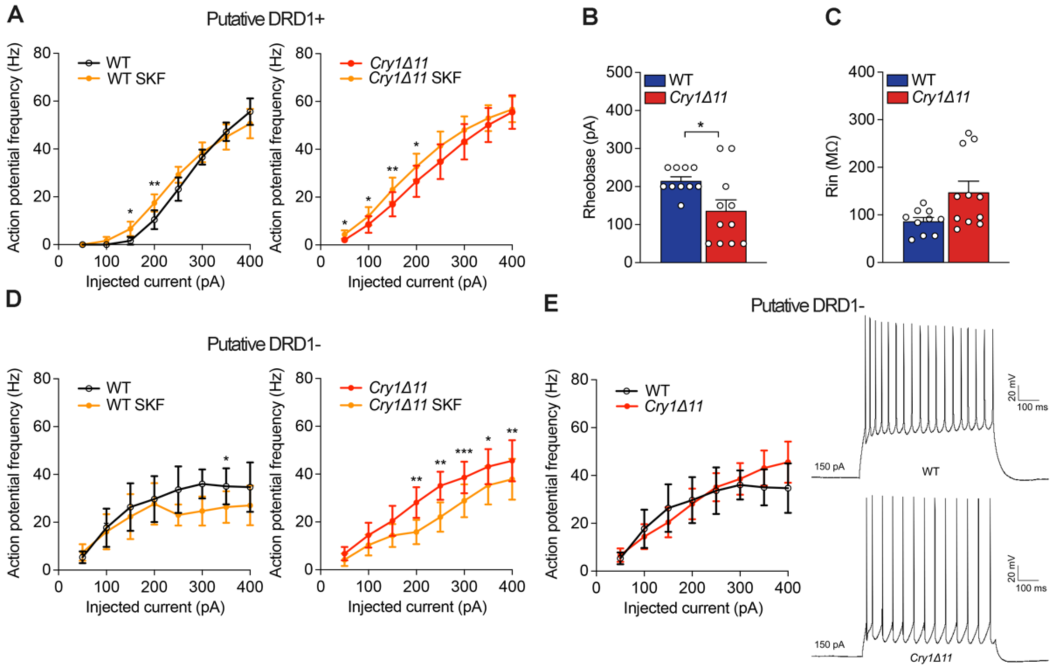

Figure S5 DRD1-MSNs in the NAc of *Cry1Δ11* mice possess more depolarized resting membrane potential and higher excitability. (A) The frequency of action potentials after DRD1 agonist SKF38393 treatment was used to identify DRD1-positive neurons. Data are presented as means ± SEM. n=10 neurons from 10 WT mice, and n=11 neurons from 10 *Cry1Δ11* mice. *p < 0.05, **p < 0.01, Repeated measure ANOVA test. (B) The rheobase of DRD1-MSNs in the NAc taken from *Cry1Δ11* mice was significantly decreased than that of WT mice. Data are presented as means ± SEM. n=10 neurons from 10 WT mice, and n=11 neurons from 10 *Cry1Δ11* mice. *p < 0.05, unpaired Student's *t* test. (C) The cell input resistance of DRD1-MSNs in the NAc was no significant between WT and *Cry1Δ11* mice. Data are presented as means ± SEM, n=10 neurons from 10 WT mice and n=11 neurons from 10 *Cry1Δ11* mice. p > 0.05, unpaired Student's t test. (D) The frequency of action potentials after DRD1 agonist SKF38393 treatment was used to identify DRD1-negative neurons. Data are presented as means ± SEM; n=10 mice/genotype; *p < 0.05, Repeated measure ANOVA test. (E)

The frequency of action potentials of DRD1-negative neurons in the NAc induced by 400 pA current steps and the representative action potentials of DRD1-negative neurons in NAc induced by 150 pA current steps. Data are presented as means ± SEM; n=10 mice/genotype; *$p< 0.05$ Repeated measure ANOVA test.

Table S1 The information for animals used in the behavioral tests

Batch	Number of animals	Zeitgeber time (ZT)	Experiments	The order of behavioral tests	Data presentation
#1	7 WT；7 *Cry1Δ11*	ZT02-04	No treatment	OFT—NOR	Figure 2-3
#2	10 WT；10 *Cry1Δ11*	ZT14-16	No treatment	—Y-maze—	Figure 2-3
#3	8 WT；8 *Cry1Δ11*	ZT14-16	Saline	LDT—EPM	Figure 6
#4	8 WT；8 *Cry1Δ11*	ZT14-16	SCH23390	—TST—FST	Figure 6

Animals were rested for 1-2 days after each behavioral test.
